# A 19-Year-Old Male With Orbital Cellulitis and Abscess Due to Fusobacterium necrophorum With Chronic Aspergillosis Resulting in Orbital Compartment Syndrome

**DOI:** 10.7759/cureus.47061

**Published:** 2023-10-15

**Authors:** Amanda Emard, Brit Long, Sara Birdsong

**Affiliations:** 1 Emergency Medicine, Brooke Army Medical Center, Fort Sam Houston, USA

**Keywords:** ocs, aspergillus, orbital cellulitis, fusobacterium necrophorum, orbital compartment syndrome

## Abstract

Orbital cellulitis is a dangerous condition that has a variety of etiologies and risk factors such as chronic sinusitis. If left untreated, it may result in orbital compartment syndrome.

A 19-year-old male presented with evidence of orbital cellulitis, increased intraocular pressures, and orbital compartment syndrome as a result of a retrobulbar abscess. He was started on ampicillin/sulbactam, the emergency clinician performed a lateral canthotomy and cantholysis, and the case was discussed with ophthalmology and otolaryngology on call. The patient was taken to the operating room for further surgical therapy. Cultures revealed Fusobacterium necrophorum and Aspergillus spp.

Orbital cellulitis is an infection of the tissue posterior to the orbital septum. Common bacterial etiologies of orbital cellulitis include Staphylococcus spp, Streptococcus spp, and Haemophilus spp. Chronic sinusitis secondary to an Aspergillus infection increases the risk of superinfection given the inability to clear nasal secretions. Diagnosis of orbital cellulitis can be clinical, but imaging with computed tomography of the orbits with intravenous contrast can assist. Treatment includes broad-spectrum antibiotics and ophthalmology consultation. If left untreated, orbital cellulitis may lead to orbital compartment syndrome, requiring lateral canthotomy and cantholysis.

Prompt identification of orbital compartment syndrome and surgical intervention with lateral canthotomy and cantholysis can help restore the function of the optic nerve if performed in a timely manner. Clinicians should consider broadening the antibiotic coverage to include carbapenems or adding on anaerobic coverage with metronidazole in patients with concern for abscess formation in the setting of chronic sinusitis.

## Introduction

Orbital cellulitis is an infection of the tissue posterior to the orbital septum. This condition is associated with a variety of etiologies, including intraorbital (endophthalmitis), exogenous (surgery, trauma, foreign body), and endogenous (bacteremia) [1]. While rare, it is associated with significant morbidity and may result in orbital compartment syndrome due to elevated intraocular pressures (IOP) [1]. This may result in permanent vision loss if the causative organisms are left untreated.

We present a case of a 19-year-old male with a history of chronic fungal sinusitis presenting with eye pain, visual disturbance, and elevated intraocular pressure ultimately diagnosed with orbital compartment syndrome associated with Fusobacterium necrophorum and Aspergillus and orbital compartment syndrome.

This case report is intended for emergency physician staff to develop a better understanding of ocular emergencies in general, but also to discuss a specific superinfection that may be present in our patient populations, requiring a need to further broaden antibiotic coverage.

## Case presentation

**Figure 1:**
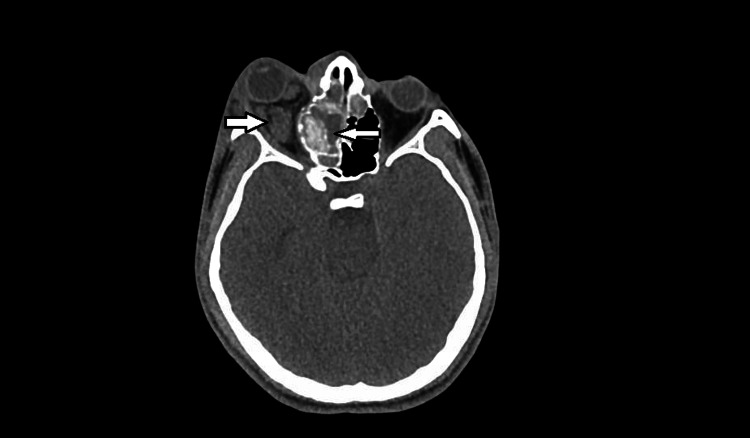
Initial CT Orbit showing sinusitis with orbital cellulitis and small fluid collection posterior to the eye without any globe deformity or ocular nerve compression.

A 19-year-old immunocompetent male with a past medical history of chronic fungal sinusitis initially presented to an outside emergency department (ED) with eye pain and blurry vision. He was noted to have significant proptosis and normal intraocular pressures on initial examination. Computed tomography (CT) orbit was obtained, which demonstrated complicated chronic sinusitis suggestive of fungal causes with remodeling of the ethmoid sinuses with post-septal interior infraorbital involvement and proptosis of the right globe (Figure 1). The patient was admitted to the hospital with acute orbital cellulitis and chronic fungal sinusitis. He was treated with intravenous ampicillin/sulbactam for four days under ophthalmology and internal medicine care, transitioned to amoxicillin-clavulanic acid without anti-fungal coverage, and discharged home given his improvement of symptoms with follow-up care scheduled in two weeks. The patient presented to the ED nine days later with increased swelling and loss of vision in the same eye upon waking that morning.

On arrival at the ED, the patient had normal vital signs and was afebrile. He displayed significant chemosis of his right eye, his eye was firm to palpation, and he had no extraocular movements. Further examination revealed IOP 52 mmHg, a fixed right pupil, and complete loss of vision.

The emergency physician discussed the case with ophthalmology and otolaryngology (ENT), administered 2 g ampicillin/sulbactam given the concern for initial spread from his sinus cavity, 4 mg morphine, timolol eye drops, and then performed an inferior lateral canthotomy and cantholysis. This resulted in minimal improvement in the IOP to 45 mmHg, and superior cantholysis was then completed within a couple of minutes of the inferior cantholysis with a repeat IOP of 42 mmHg.

**Figure 2:**
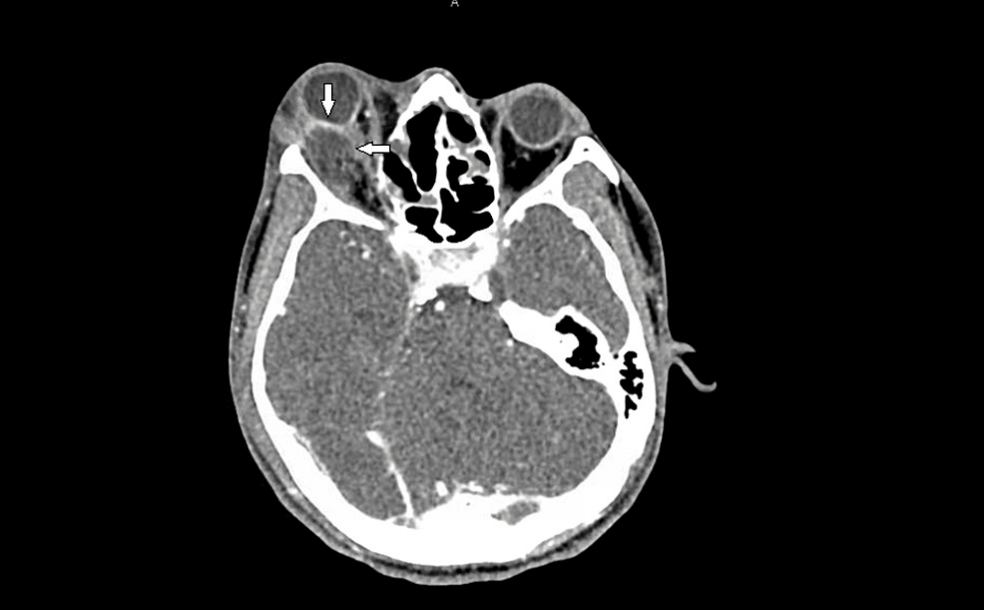
CT Orbit from the second visit showing an axial view with a right convex orbital abscess arising from the lateral wall of the orbit and obscuring the view of the lateral rectus with deformity noted to the globe and compression of the optic nerve.

Repeat CT orbits (Figure 2) post-cantholysis showed marked progression of the right orbital cellulitis with large inferior post-septal orbit abscesses and large lateral posterior orbital phlegmon. There was also marked right proptosis with deformity of the right globe and optic nerve suggestive of orbital compartment syndrome.

The patient was taken to the operating room emergently with ophthalmology for abscess drainage and globe exploration and was admitted to an internal medicine service for further care. Bacterial cultures from initial incision and drainage were positive for Fusobacterium necrophorum and Aspergillus (no species isolated) without any available drug sensitivities. The patient subsequently underwent three incision and drainage procedures over the next six days due to continuing abscess formation and worsening of eye pressures. The patient was discharged after improvement following multiple surgical interventions. Follow-up visits in the outpatient clinic report that vision was not able to be restored to the affected eye.

## Discussion

Ocular complaints account for a significant number of ED visits, with approximately two million patient visits per year. Up to 41.2% of these eye-related concerns are emergent based on the International Classification of Diseases (ICD)-9 codes at the time of discharge [2].

Orbital cellulitis develops from an extension of infection, usually from the sinus cavities, into the area around the orbit of the eye. Etiologies include intraorbital (endophthalmitis), exogenous (surgery, trauma, foreign body), and endogenous (bacteremia) [1]. The most common bacterial organisms include Staphylococcus spp, Streptococcus spp, and Haemophilus spp [3]. Presenting signs and symptoms of orbital cellulitis typically include changes in vision, proptosis of the affected eye, peri-orbital erythema, and pain with extraocular eye movement. Diagnosis of orbital cellulitis can be made clinically. Laboratory analysis is not definitive but may reveal elevated white blood cell count with a left shift and inflammatory markers [e.g., c-reactive protein, erythrocyte sedimentation rate (ESR), procalcitonin], but these should not be used to exclude the diagnosis. CT of the orbits with IV contrast can assist with diagnosis, as can magnetic resonance imaging if available. Ultrasound has been evaluated, though data are inconclusive regarding sensitivity and specificity [1]. If orbital cellulitis is diagnosed, a third-generation cephalosporin (e.g. ceftriaxone) plus metronidazole, ampicillin-sulbactam, or piperacillin-tazobactam, in addition to vancomycin are the preferred broad-spectrum antibiotics [3]. Ophthalmology and otolaryngology (ENT) consultation is also recommended due to the need for emergent surgical management in most of these cases. Ophthalmological procedures often include lateral cathotomy and cantholysis, with or without bony orbital decompression, if not already performed in the emergency department [4]. If these interventions do not result in improvement, the patient often requires surgical decompression under general anesthesia to ensure full decompression of the bony orbit and globe [4]. While this is primarily of ocular concerns, if the area of involvement is diffuse or requires medial components of the orbit, it is recommended that ENT be consulted for appropriate management [4]. ENT has the ability to often perform endoscopic drainage of these abscesses to provide source control when ophthalmology may not be available [4].

While bacterial causes are the most common cause of orbital cellulitis, fungal etiologies are possible, with Aspergillus being the most common fungal isolate from sinusitis [5]. As demonstrated in the discussed case of bacterial superinfection, the patient was diagnosed with chronic fungal sinusitis, or fungal rhinosinusitis, previously. Fungal sinusitis associated with Aspergillus is most common in warmer, humid climates [5]. In immunocompromised patients, Aspergillus can cause invasive infection of the lungs or sinuses, but invasive infection in otherwise healthy patients is rare [5]. Allergic fungal rhinosinusitis (AFRS) in the immunocompetent patient is a generally mild condition that accounts for 5-10% of all cases of chronic sinusitis, as in our patient [6]. AFRS typically results in chronic edema of the paranasal sinuses with an inability to clear nasal secretions effectively, which may lead to bacterial superinfection [6].

Fusobacterium necrophorum is an emerging pathogen resulting in otogenic and paranasal sinus infections [7]. F. necrophorum is an anaerobic gram-negative rod found among the respiratory, gastrointestinal (GI), and female urogenital tracts [7]. This bacteria has been implicated previously in post-anginal sepsis, Lemierre’s syndrome, mastoiditis, meningitis, and sinus thrombosis [7]. Prior case report literature describes complications of F. necrophorum to include Lemierre’s syndrome and Pott puffy tumor, but to date, there have been only a handful of documented cases associated with orbital compartment syndrome [4]. Identification of the bacteria is often delayed with limited ability to detect the isolate which often results in poor patient outcomes in the documented cases [7]. In the patient reports reviewed, patients often had improvement after being started on a carbapenem, but it was often identified too late in the patient course [7]. Antibiotic therapy should be broad spectrum to include anaerobic coverage, including a carbapenem, piperacillin/tazobactam, or ceftriaxone plus metronidazole [8].

If untreated, orbital cellulitis can extend into surrounding tissues and lead to an abscess. This abscess in the tight retrobulbar space may increase IOP, resulting in orbital compartment syndrome (OCS) [1]. Ocular compartment syndrome is a surgical emergency that can lead to vision loss if not promptly treated. Patients present with proptosis, chemosis, restricted extraocular movements, and resistance to retropulsion on ocular examination. OCS is a clinical diagnosis with IOP measurement and does not require any further imaging. IOPs 30 mmHg or greater are concerned for increased intraocular pressure [4]. If identified, patients with OCS and an IOP of greater than 40 mmHg require emergent surgical decompression with a lateral canthotomy and cantholysis of the inferior crus first, with repeated measurement of intraocular pressures. If ocular pressures do not return to baseline, cantholysis of the superior crus should be performed. Literature suggests that irreversible damage occurs to the ocular nerve after 105 minutes of associated compression [4]. Adjunctive medical therapy may include corticosteroids, carbonic anhydrase inhibitors like acetazolamide or brinzolamide, osmotic agents, and aqueous suppressants [4].

## Conclusions

Patients with chronic sinusitis are at risk of bacterial superinfection with atypical bacteria such as Fusobacterium necrophorum. This can lead to the development of orbital cellulitis with abscess and OCS in an otherwise healthy, immunocompetent patient. OCS is caused by mass effect causing increased ocular pressures due to the confined retroorbital space. Signs of symptoms of orbital cellulitis include eye pain, vision changes, proptosis, and restricted extraocular movements. Treatment includes broad-spectrum antibiotics and ophthalmology consultation. If untreated, this may lead to OCS. Management of OCS includes lateral canthotomy and cantholysis and emergent ophthalmological consult.
